# Innovation signals: leveraging machine learning to separate noise from news

**DOI:** 10.1007/s11192-023-04672-y

**Published:** 2023-04-12

**Authors:** Christian Mühlroth, Laura Kölbl, Michael Grottke

**Affiliations:** 1 Department of Statistics & Econometrics, Friedrich-Alexander-Universität Erlangen-Nürnberg, Lange Gasse 20, 90403 Nuremberg, Germany; 2 Global Data Science, GfK SE, Sophie-Germain-Straße 3–5, 90443 Nuremberg, Germany

**Keywords:** Weak signals, Strong signals, Corporate foresight, Innovation management, Machine learning, Artificial intelligence, Trend scouting, Technology scouting, Startup scouting, 68T05, 68T50, 62H30, C8, C88, E17, M1, M19, O31, O32, O33

## Abstract

The early detection of and an adequate response to meaningful signals of change have a defining impact on the competitive vitality and the competitive advantage of companies. For this strategically important task, companies apply corporate foresight, aiming to enable superior company performance. With the growing dynamics of global markets, the amount of data to be analyzed for this purpose is constantly increasing. As a result, these analyses are often performed with an unreasonably high investment of financial and human resources, or are even not performed at all. To address this challenge, this paper presents a machine-learning-based approach to help companies identify early signals of change with a higher level of automation than before. For this, we combine a newly-proposed quantitative approach with the existing qualitative approaches by Cooper (stage-gate model) and by Rohrbeck (corporate foresight process). After a search field of interest has been defined, the related data is collected from web news sites, early signals are identified and selected automatically, and domain experts then assess these signals with respect to their relevance and novelty. Once it has been set up, the approach can be executed iteratively at regular time intervals in order to continuously scan for new signals of change. By means of three case studies supported by domain experts we demonstrate the effectiveness of our approach. After presenting our findings and discussing possible limitations of the approach, we suggest future research opportunities to further advance this field.

## Introduction

Global and digital trade have made markets become increasingly dynamic over time. Reduced market entry barriers encourage existing companies and startups to enter markets significantly faster than before, technological disruption transforms entire markets in even shorter cycles, and rapidly-changing customer needs constantly lead to new products, services, and business models (Utterback et al., 2019).

The recent past has shown that failing to react properly to the accompanying technological and social change bears a major risk for companies to lose large proportions of their market share (Lucas & Goh, 2009) or sometimes even to lose their entire economic viability (Weber, 2016; Mühlroth et al., 2021). In contrast, those companies which obtained the ability to effectively identify, assess, and interpret these signals were found to be more likely to deliver successful innovations, to outperform other market players, to attain superior profitability, and to gain superior market capitalization growth (Rohrbeck et al., 2015; Rohrbeck & Kum, 2018). Therefore, companies have a strong need to constantly increase both their awareness for and their responsiveness to external change by scanning their corporate environment for early and relevant signals of change (de Geus, 1997; von der Gracht et al., 2010; Durst & Durst, 2016). However, in most cases these signals are detected by chance rather than by a systematic approach, exposing companies to a risk that could well be prevented (Rohrbeck & Bade, 2012; Mühlroth & Grottke, 2022).

As market dynamics and thus complexity increases, the amount of data that can be analyzed towards early signals of change is growing, too. Typical information of interest includes new and previously-unknown but relevant topics, inspirations on how market trends and technology trends are being addressed by companies and institutions, activities that indicate where competitors are heading, as well as news on startups, mergers, and acquisitions (Keller & von der Gracht, 2014). As a result, growing interest in the use of data analytics and machine learning to discover this information can be observed both in research and in practice (Mühlroth & Grottke, 2018; Kölbl et al., 2019; Schuh et al., 2021).

A recent literature review shows that existing research in this area has addressed the challenge from different perspectives (Mühlroth & Grottke, 2018). Rohrbeck et al. (2006) and Thom et al. (2010) describe how to leverage international scouting networks, entirely based on human interaction and knowledge, in order to identify technological developments and trends. Other approaches discover meaningful topics in large data collections of unstructured text, image, or video data (Karl et al., 2015; Madani, 2015; Li et al., 2016; Mryglod et al., 2016), detect anomalous patents given a predefined technology area (Yoon & Kim, 2012; Barirani et al., 2013; Carley et al., 2018), or validate human-made hypotheses about possible future developments (Thorleuchter et al., 2014). Previous research also aims to predict the future development of detected weak signals (Gutsche, 2018), to analyze scientific publications and patents (Garner et al., 2017) or online communities in social media (Eckhoff et al., 2015) for emerging terms, to detect risks such as the COVID-19 pandemic early on using Latent Dirichlet Allocation (El Akrouchi et al., 2021), and to perform statistical trend analysis (Cataldi et al., 2013; Ena et al., 2016).

However, existing approaches often still require a high degree of involvement of human experts (Holopainen & Toivonen, 2012; El Akrouchi et al., 2021). While promising data-driven approaches do exist (Thorleuchter & van den Poel, 2013; Eckhoff et al., 2014; Garcia-Nunes & da Silva, 2019), they usually process a very large amount of data without separating the noise from the actual news. As a result, they risk spamming their users with many signals. Existing approaches are also prone to cognitive issues (e.g., the human experience bias) due to a lack of automation; moreover, they are often built upon static data collections and are thus not designed to adapt to the ever-changing information in the corporate environment of companies (Mühlroth & Grottke, 2018).

Therefore, this paper aims to address the aforementioned issues in research and practice by offering the following contributions: First, we present the theoretical framework which guides our work in the section “Theoretical framework” and discuss the research method applied in section “Research method”. Second, in the section “Innovation signals approach”, we propose a new approach for leveraging machine learning to continuously detect and respond to signals of change for corporate strategy, foresight, and innovation processes. We hereby aim at embedding a-priori human mental models (Cawley & Talbot, 2010; Palomino et al., 2013; Milanez et al., 2014) to a lesser degree and to thus reduce the impact of the human experience bias. We then apply our approach in three case studies in the section “Case studies” to demonstrate its effectiveness in practice, and we present the results of a sensitivity analysis regarding the clustering method chosen in the section “Comparison of clustering results for different linkage methods”. Next, in the section “Discussion” we derive implications for research and practice, we discuss some of the limitations of our approach, and we suggest future research opportunities for further advancing this field. Finally, section “Conclusion” concludes our paper.

## Theoretical framework

For several decades, foresight has been applied to identify, observe, and interpret external change in the corporate environment in order to develop appropriate tactical and strategic responses (Ansoff, 1975; Rohrbeck et al., 2015). Its outcomes are further processed in related business processes such as in strategy and innovation processes (Rohrbeck et al., 2006; von der Gracht et al., 2010; Heinonen & Hiltunen, 2012; Mühlroth et al., 2021).

From early on, environmental scanning has been used as a central activity for both the identification and the observation steps in foresight (Aguilar, 1967). The changes identified have been categorized according to their main driver, i.e., social, technological, economic, environmental, and political (also abbreviated as *STEEP*; Hiltunen, 2008a; Carr & Nanni, 2009; Durst & Durst, 2016; Wiser et al., 2019). These categories provide the framework in which companies are to expect external influences that they can only rarely (if at all) influence by themselves. In addition, these influences are differentiated according to their direction of impact: market pull (sometimes also called demand pull) and technology push. The former refers to (sometimes hidden) needs and requirements for a new solution to a (sometimes unknown) existing problem (“problem seeks solution”), whereas the latter refers to an innovative technology that is first developed and then applied to a product or service (“solution seeks problem”;  Brem & Voigt, 2009; Di Stefano et al., 2012).

The purpose of environmental scanning is thus to observe each category and each direction of impact, in order to detect signals of change at an early stage. The information to be gained is classified into different levels of maturity, such as weak signals, strong signals, and (mega) trends (Holopainen & Toivonen, 2012):

- *Weak signals* are considered early signs of potentially-imminent and impactful emerging issues and potential future changes (Ansoff, 1975; Kuosa, 2010). The information is still too noisy, incomplete, imprecise, and unconfirmed to allow an accurate assessment of its impact and to develop an adequate response (Saritas & Smith, 2011; Mendonça et al., 2012). Over time, weak signals may either turn out to be false signals or develop into strong signals. Therefore, weak signals should be monitored for their future evolution (Hiltunen, 2010).
- *Strong signals* emerge if similar weak signals are pointing in the same direction. If the signal is found in different sources over a short period of time, this indicates that the formerly weak signal has received increasing attention and has thus become stronger (Hiltunen, 2010). Strong signals are considered to be sufficiently visible and concrete (Ansoff & McDonnell, 1990), and the knowledge about them is more extensive; i.e., more information on the primary driver of change (e.g., social, technological, or environmental) and the direction of impact (e.g., market pull) has become available (Kuosa, 2010). Additional information, such as the source, shape, and sense of the strong signal evolving into a potential opportunity or threat can be collected, response strategies can be defined, and their outcomes can be estimated (Uskali, 2005).
- *Trends and mega trends* have the highest level of maturity and are described as long-term developments, arising from generalizable change and innovation (Saritas & Smith, 2011). Detecting such a development requires a considerable amount of data (Mühlroth & Grottke, 2018). In contrast to weak and strong signals, trends spread more slowly, but their pervasiveness is much higher. Moreover, trends often have a global reach, and both companies and institutions do not any longer have any considerable influence on them (Hiltunen, 2008b).

Following this terminology, weak and strong signals refer to an early stage, whereas trends and mega trends describe developments which have already been happening for a longer period of time. While weak signals do not contain sufficient information to be assessed and interpreted (Ansoff, 1975; Hiltunen, 2010; Saritas & Smith, 2011), exclusively focusing on (mega) trends may result in detecting developments too late to take any effective strategic measures in response (Mühlroth & Grottke, 2018). We therefore propose a new approach that aims at identifying strong signals. It tries to automatically detect and report such strong signals early on before they possibly develop into a trend. This leaves enough time to explore potential opportunities (e.g., new and innovative products for changing customer needs), as well as to address possible risks (e.g., a changed competitive situation due to new market entrants) arising from the imminent change (Rohrbeck & Kum, 2018; Gordon et al., 2020).

Moreover, our approach aims at increasing the degree of automation of these activities, and at preventing human experts from being spammed with too much irrelevant information. Thus, they will be enabled to invest more time in interpreting and responding to the strong signals, rather than identifying them.

## Research method

As defined in the previous section, early signals of change have the inherent ability to impact future developments. The verification of an approach which aims at identifying such signals before their effects are visible requires knowledge about the future.

One way to validate such an approach is to conduct a retrospective study against a well-known reference benchmark (Mills et al., 2010). To do so, we would have to select (mega) trends observable in the present and then apply our proposed approach to data from the past. If we then discover traces of early signals which, assuming causality, have led to these trends, we could claim that our proposed approach is effective.

However, the identification of early signals does not depend on whether or not they will actually *have* an impact in the future, but rather on their *potential* to do so (Kölbl & Grottke, 2019). A retrospective approach can confirm the detection of a specific signal that has indeed developed into a trend; however, it cannot account for those signals which had the same potential but have not lived up to it. Thus, the future development of such an early signal does not determine its nature as a true or false signal. Moreover, such a retrospective study may be subject to a strong look-ahead bias: the knowledge of the trends selected might influence the decisions made in preparing the study, e.g., the search field and the selected time period of the data collection.

Based on these considerations, we do not carry out a retrospective study to evaluate our approach. Rather, we have decided to validate it with expert evaluations by means of three case studies (Yin, 2018). In each case study, early signals of change are identified and are then evaluated by experienced domain experts against two criteria: relevance to their business and novelty to them as an expert. This method allows us to classify whether or not an identified signal has the potential to have an impact on the future (Kölbl & Grottke, 2019), and we are thus able to measure the percentage of true signals within the identified ones. Moreover, it avoids the risk of the look-ahead bias. The expert ratings are then used to study the effectiveness and efficiency of the approach.

It should thus be noted that while the evaluation conducted by the experts is part of our proposed overall approach, the part of our approach that is validated in it is the previous automated task of identifying strong signal candidates.

## Innovation signals approach

As the basis for our approach we employ the four-step process described by Rohrbeck et al. (2006) as well as by Boe-Lillegraven and Monterde (2015), since it is widely used in practice, and we adapt it to our context. The four steps are: (1) the identification step, where new trends and technologies are identified; (2) the selection step, where the most-promising new trends and technologies are selected; (3) the assessment step, where the results are evaluated by experts in greater detail; and (4) the dissemination step, where key information about the results is aggregated for presentation to the management.

In our approach, we focus on the automation of the first two steps (*identification* and *selection*) and on the third step to evaluate the automated steps by experts (*assessment*), while we consider the fourth step (*dissemination*) as a separate stage where the results need to be communicated to other interested stakeholders.

Furthermore, we structure these steps following Cooper’s stage-gate model (Cooper et al., 1986). It is considered as best practice (Müller-Wienbergen et al., 2011), and it divides each process step into the sub-steps *stage* (where one or more activities are performed to generate a long list of new insights) and *gate* (where the insights generated are combined, condensed, assessed, prioritized, and filtered to a short list). Thus, after each gate a result becomes available, which is further processed in the following stage. More precisely, we adjust the gates from Cooper’s model and turn them into filters: at each gate, it is decided if a data point is interesting enough to pass to the next stage. The model thus allows us to present the various decision points of our approach in a transparent manner.

**Fig. 1:**
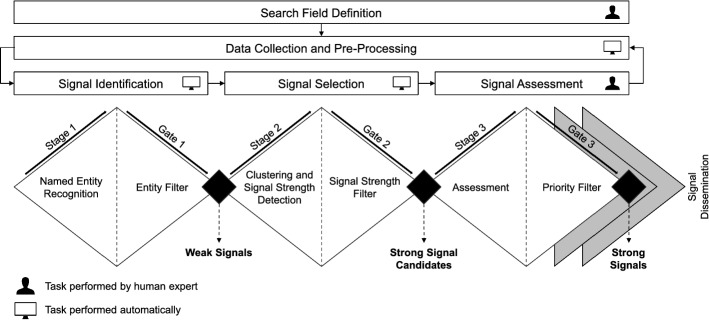
Overview of the innovation signals triple diamond model

Finally, we visualize the process steps by adapting the double diamond model (Howard et al., 2008). The dichotomy of each diamond in terms of divergent and convergent thinking relates to the stage and gate of each sub-step, respectively, which results in the triple diamond model visualized in Fig. 1.

After the search field has initially been defined, the respective data is automatically collected and pre-processed. In this data, weak signals are detected during the *signal identification* step. During the *signal selection* step, strong signal candidates are identified, which are then interpreted to obtain the final strong signals during the *signal assessment* step. Subsequently, the resulting strong signals are disseminated and further processed in foresight, strategy, and innovation processes (Brem & Voigt, 2009; Thom et al., 2010; Di Stefano et al., 2012; Barbieri & Álvares, 2016; Durst & Durst, 2016).

Within these steps, our proposed approach makes use of advanced machine learning techniques that are combined in such a way that a high degree of automation and repeatability is achieved. Human expert assistance is only required for the initial search field definition and for the assessment of the results, whereas both weak signals and strong signal candidates are detected automatically. Furthermore, our approach allows to collect new data at predefined time intervals (e.g., daily, weekly, or monthly), thus enabling a continuous monitoring for any number of search fields of interest. Each step and its working principles are further described in the following subsections.

### Search field definition

The starting point of our proposed approach is the *search field definition*. A search field describes a specific field of interest, e.g., a specific technological field in which early signals are to be scouted and monitored (Keller & von der Gracht, 2014; Rohrbeck, 2014).

First, a search query is defined together with human expert support in order to reduce the available data to a subset of interest. It is defined by a list of relevant keywords with optional wildcards relevant for the given search field, concatenated by Boolean operators. Second, one or more appropriate data sources are selected (e.g., websites, webnews, or weblogs; Mühlroth & Grottke, 2018). Finally, the preferred start date (i.e., the earliest date of publication of the data) and the preferred time interval for data updates (e.g., daily, weekly, or monthly) are specified. Following this approach, multiple search fields can be defined.

### Data collection and pre-processing

During *data collection*, the search query is used to retrieve the data from the selected data sources. Each element obtained (e.g., one news article) will be referred to as a “document”, and the set of all documents for a given search field will be referred to as the “corpus”. In addition to the title and the content of each document, we also store additional meta data, such as its publication date and its original source (in terms of its uniform resource identifier; URI) for later analysis.

The data collected is then sent to further *data pre-processing* in order to increase the quality of the subsequent data analysis (Thorleuchter & van den Poel, 2013). So far, no universally-accepted standard has been established for this purpose, and human experts are often still required to iteratively refine intermediate results (Mühlroth & Grottke, 2018). In our approach, however, we have employed a fully-automated data pre-processing pipeline:

At first, we clean the data collected by discarding documents with either no title or no content (since we consider them to be data errors), and by removing all digit-only characters, special characters, diacritics, and markup tags. We then remove terms with less than two or more than thirty characters (as we consider them to be leftovers, garbled text, or an URI string; Karl et al., 2015), as well as remaining multiple whitespaces. Finally, we transform the cleaned documents to lowercase (Thorleuchter & van den Poel, 2013). Since the individual words in the documents are still separated by whitespaces, we apply tokenization to chunk the text; each token now represents a word that consists of alphanumeric characters. We then apply part-of-speech (PoS) tagging to the tokens generated and only keep proper nouns (such as “usa” or “google”), nouns (such as “intelligence” or “robot”), verbs, adjectives, and numerals, while we discard tokens with any other PoS tag. The remaining tokens are further lemmatized to transform both derivationally-related and inflectional words back to their common base form.

Next, we apply stop word filtering to remove commonly-used words (such as “have”, “to”, or “often”), as these words are expected to contribute only little or even no information gain. For this, we have lemmatized and combined stop word lists from different sources to remove as many stop words as possible.

We further transform the remaining tokens into *n*-grams (i.e., tokens of length *n*, such as “artificial intelligence” for $n=2$), which have been found to improve the quality of text mining results (Tan et al., 2002; Lau et al., 2013). Some implementations of this transformation form an *n*-gram only if it is recognized as a phrase (based on a phrase detector or a dictionary), and they then discard its components of length $n-1$ or less. Table 1 shows an excerpt taken from the English Wikipedia article on artificial intelligence before and after pre-processing with $n=2$. Note that the resulting document contains the bi-gram “computer_science”, while the two uni-grams “computer” and “science” have been discarded.

**Table 1:** Example document before and after pre-processing

Before pre-processing	In computer science, artificial intelligence (AI), sometimes called machine intelligence, is intelligence demonstrated by machines, in contrast to the natural intelligence displayed by humans. Colloquially, the term “artificial intelligence” is often used to describe machines (or computers) that mimic “cognitive” functions that humans associate with the human mind, such as “learning” and “problem solving” [2]. A quip in Tesler’s Theorem says “AI is whatever hasn’t been done yet [4].”
After pre-processing	[“computer_science”, “artificial_intelligence”, “call”, “machine_intelligence”, “intelligence”, “demonstrate”, “machine”, “contrast”, “natural_intelligence”, “display”, “human”, “term”, “artificial_intelligence”, “machine”, “computer”, “mimic”, “cognitive_function”, “human”, “human_mind”, “learning”, “problem_solving”, “quip”, “tesler”, “theorem”]

Finally, we create the document-term matrix (DTM) representing documents in a vector space model (VSM). The $d^{th}$ row of the DTM is a vector $\varvec{w}_d$ indicating which terms are contained in document *d*. Rather than simply using zeros and ones, we apply the TF-IDF model (Robertson, 2004; Murray & Renals, 2007; Rehurek & Sojka, 2010) for weighting the individual terms. The $t^{th}$ entry in $\varvec{w}_d$, the weight $w_{d,t}$ of term *t* in document *d*, is computed by multiplying a local component (i.e., term frequency; TF) with a global component (i.e., inverse document frequency; IDF):

1 $$\begin{aligned} w_{d,t} = tf_{d,t} \cdot \log _2\left( \frac{D}{df_t} \right) , \end{aligned}$$

where $tf_{d,t}$ is the frequency (i.e., raw count) of term *t* in document *d*, $D=|\mathcal {D}|$ is the total number of documents in corpus $\mathcal {D}$, and $df_t$ is the number of documents that contain the term *t* (i.e., document frequency). With this weighting scheme, a term is rated highly if it occurs often within a particular document but only rarely across the set of all documents (Murray & Renals, 2007).

In addition, the weights are normalized using cosine normalization:

2 $$\begin{aligned} \varvec{w}^*_{d} = \frac{\varvec{w}_{d}}{\parallel \varvec{w}_{d} \parallel }. \end{aligned}$$

By making use of *n*-grams (and by applying Named Entity Recognition, see in the section “Signal Identification”), we go beyond the simple bag-of-words approach which only accounts for the unordered list of individual words. While even newer approaches based on artificial neural networks are superior in use cases where semantics play an important role (Mikolov, 2012), they are more complicated to implement and computationally more expensive during runtime (e.g., distributed representations of sentences and documents as proposed by Le and Mikolov, 2014). For our purpose of detecting topics in documents from websites, news, or weblogs at an early stage in an automated way, we expect a high data volume per day, especially if multiple search fields are to be observed simultaneously. We therefore need an inexpensive and computationally efficient model that does not require any upfront training. Furthermore, we believe that information on semantics is negligible in our case, since we want to search for documents that are highly similar in content and group them into clusters. The VSM approach chosen thus seems to provide the necessary characteristics for our use case.

### Signal identification

The pre-processed and vectorized documents are analyzed towards weak signals in the *signal identification* step. Its goal is to automatically separate possible signals of change from less relevant information.

For this, we apply Named Entity Recognition (NER), a technique for Natural Language Processing (NLP). NER makes it possible to extract entities from unstructured text data and to label them with specific entity types (Nadeau & Sekine, 2007), such as “ORG” (for organizations), “PERSON” (for persons), or “GPE” (for locations). In the example document from Table 1, the token “tesler” would be annotated with the label “PERSON”, as it refers to Larry Tesler, who coined the said quote (Hofstadter, 1980).

Since weak signals are suspected to be some new information sent by or about someone (i.e., a natural or legal person; Hiltunen, 2008a), in our approach we only keep those documents in which at least one organization or one person is mentioned. Those documents are considered to be weak signals and are further processed in the subsequent steps, whereas all other documents are discarded from the document collection.

### Signal selection

The next step *signal selection* aims at detecting candidates for strong signals. According to the definition by Hiltunen (2010), mentioned in the section “Theoretical framework”, a strong signal emerges if weak signals are pointing in the same direction. Since we have previously projected the documents into the VSM (see in the section “Data collection and pre-processing”), we can now group those document vectors that actually point in the same (or at least in a very similar) direction.

In our approach we make use of clustering, an unsupervised machine learning technique to aggregate similar weak signals. Since their number can be very high, we apply agglomerative (also called “bottom-up”) hierarchical clustering, which does not require the number of clusters to be specified upfront (Rokach & Maimon, 2005). At the start of this algorithm, the normalized weight vector $\varvec{w}^*_{d}$ of each document *d* is contained in its own cluster $C_d$. In every iteration of the algorithm, the pairwise distances of all cluster combinations are calculated, and the two clusters with the smallest distance are merged. This procedure is repeated until a predefined termination criterion is reached.

As we want documents that are very similar or even identical to be clustered together, we use Ward’s method for the distance measure since it minimizes the total within-cluster variance (Everitt et al., 2011). It defines the distance to be minimized as the merging costs of two clusters in terms of the increase in total within-cluster variance after merging them (Pedregosa et al., 2011):

3 $$\begin{aligned} dist(C_i, C_j) = \frac{|C_i|\cdot |C_j|}{|C_i|+|C_j|} \parallel \bar{\varvec{c}_i} - \bar{\varvec{c}_j} \parallel ^2, \end{aligned}$$

where $|C_i|$ is the number of documents in cluster *i*, and $\bar{\varvec{c}_i}$ is its center,

$$\begin{aligned} \bar{\varvec{c}_i} = \frac{1}{|C_i|} \sum _{\varvec{w^*_d}\in C_i} \varvec{w^*_d}. \end{aligned}$$

The custom termination criterion in our approach is based on a threshold ${\alpha }_z$. It is determined as the *z* quantile of the $m={D \atopwithdelims ()2}$ pairwise distances between all initial clusters $C_1, \ldots , C_D$. Let $dist_{(i)}$ denote the $i^{th}$ order statistic of these *m* distances, i.e., $dist_{(1)} \le dist_{(2)} \le \ldots \le dist_{(m)}$. For a given $z \in [0,1]$ and based on these values, the threshold is calculated as

4 $$\begin{aligned} {\alpha }_z = dist_{(\lfloor h \rfloor )} + (h - \lfloor h \rfloor ) \cdot ( dist_{(\lfloor h \rfloor + 1)} - dist_{(\lfloor h \rfloor )}), \end{aligned}$$

where $h = (m-1) z +1$, and $dist_{(m+1)}$ is an arbitrary finite value, such that ${\alpha }_1 = dist_{(m)}$. In other words, if $z=(i-1)/(m-1)$ with $i \in \{1, 2, \ldots , m\}$ then the threshold/quantile is simply the $i^{th}$ order statistic $dist_{(i)}$; otherwise, it is calculated based on a linear interpolation between two subsequent order statistics.

Denote the clusters in the set $\mathcal {C}^u$ obtained in the $u^{th}$ iteration of the algorithm by $C^u_1, C^u_2, \ldots ,$ $C^u_{D-u}$. If the distances between all  pairs of these clusters are greater than ${\alpha }_z$, then the termination criterion is reached and the clustering stops. We refer to the number of iterations after which the algorithm terminates as $u^*$.

A second indicator for the emergence of a strong signal, besides the existence of several weak signals pointing into the same direction, is when these weak signals are coming from different sources (see in the section “Theoretical framework”). We therefore rank the clusters obtained after the algorithm has terminated by the count of their respective unique document sources (i.e., distinct website domains). To do so, we extract the source $s_{c,d}$ of each document *d* in cluster $C_c^{u^*}$ ($c=1, \ldots , D-u^*$), and add it to a set $\mathcal {S}_c$; in this set, each distinct source can only occur once. We then interpret the cardinality of $\mathcal {S}_c$ (i.e., the number of different sources) as the strength of the signal represented by cluster $C_c^{u^*}$:

5 $$\begin{aligned} strength_c = |\mathcal {S}_c|. \end{aligned}$$

In order to automatically select those signals which should be passed on to the next step, we calculate a threshold value ${\beta }$, which is simply the arithmetic mean over all the values for signal strength:

6 $$\begin{aligned} {\beta } = \frac{1}{D-u^*} \sum _{c=1}^{D-u^*} strength_c. \end{aligned}$$

Those clusters whose signal strength is above average, i.e., which satisfy the condition $strength_c > {\beta }$, pass the gate of the current signal selection step and are considered as strong signal candidates.

Clearly, using the median rather than the arithmetic mean would always leave us with half of the previously-remaining signals. However, we expect the distribution of signal strengths to be highly right-skewed, with few signals having very high strength values. As such outliers are boosting the arithmetic mean, our approach is likely to discard a substantial part of the signals that had remained prior to this step, leaving us with a smaller amount of signals.

These strong signal candidates are presented to the human experts in the next step.

### Signal assessment

In the *signal assessment* step, each one of the strong signal candidates identified in the previous step is presented to the human experts; in addition to a topic name (for which the title of the news article most strongly associated with the cluster is chosen), a list with links to all the news articles included in the cluster is shown. For assessing the strong signal candidates, two evaluation criteria are applied, namely relevance (“How relevant is this information to our company and business?”) and novelty (“How new is this information to us?”) on a dichotomous scale with categories “low” and “high”.

**Fig. 2:**
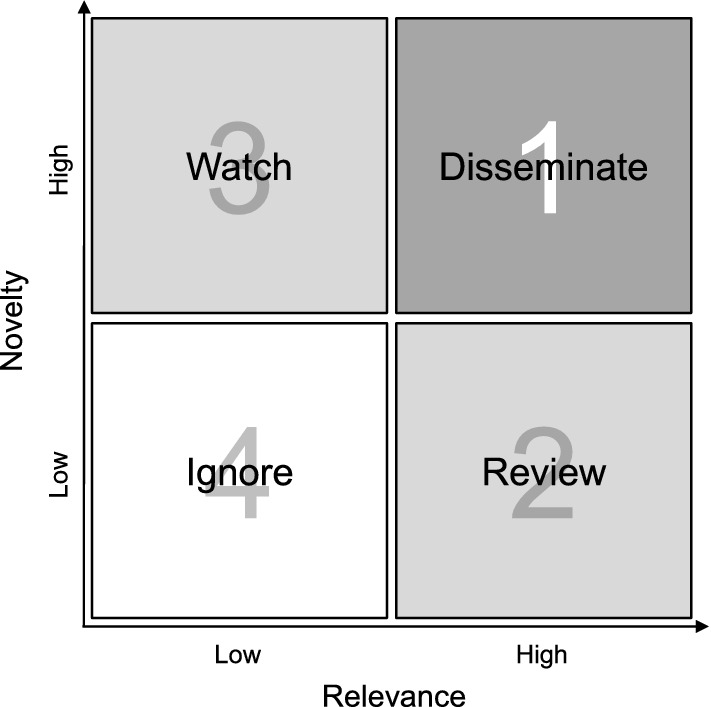
Relevance-novelty prioritization matrix

Based on the experts’ assessment, a 2-by-2 prioritization matrix for the strong signal candidates is built as shown in Fig. 2. According to their classification into one of the four quadrants, we give the following recommendations for action:

1. A signal with high relevance and high novelty qualifies as a strong signal. It is advisable to disseminate it as soon as possible to related business processes such as strategy making, foresight, and innovation, in order to explore its potential impact on the company and the business.
2. If a signal has high relevance but low novelty, then the information is already known to the company. Since its relevance is still high, it is recommended to verify whether and how the signal has been processed internally and to re-emphasize it if necessary. In addition, the environment surrounding this signal should be monitored closely to detect changes in this area at an early stage.
3. A signal with low relevance but high novelty may be deprioritized for the time being, but it should be watched closely, as even small changes in this area have the potential to make the signal more relevant.
4. A signal with low relevance and low novelty is considered as noise, and it should therefore be ignored.

Since our proposed approach collects new data and automatically generates further strong signal candidates at regular time intervals (see Fig. 1), companies are enabled to monitor and prioritize changes in their corporate environment on a continuous basis.

## Case studies

We have implemented our proposed approach combining different machine learning techniques in Python. The implementation makes use of the libraries NumPy (Oliphant, 2006), Pandas (McKinney, 2010), spaCy (Honnibal & Johnson, 2015), gensim (Rehurek & Sojka, 2010), and scikit-learn (Pedregosa et al., 2011).

For stop word filtering we have combined and lemmatized stop word lists from the Python NLTK package (Honnibal & Johnson, 2015) and from Oracle’s open-source database MySQL. When parameterizing our approach, we set $n=2$ for the *n*-gram generation, since especially bi-grams have been found to improve the quality of results in text mining (Tan et al., 2002; Lau et al., 2013). For this transformation we make use of the implementation available in the gensim library. In cases where this module recognizes a phrase, it keeps that phrase as a bi-gram and discards the two uni-grams. To this end, we train the phrase detector based on the respective corpus generated (i.e., the set of all documents for a given search field). Moreover, we apply the pre-trained transition-based NER component of SpaCy, an open-source software library for advanced NLP (Honnibal & Montani, 2017), to annotate our documents and to thus obtain additional meta information about their content. Using deep learning, this component has been specifically tuned for written text from the web (e.g., webnews and weblogs) and it therefore achieves high accuracy scores for this type of data. We further set $z=0.05$ for the termination criterion for hierarchical clustering.

In the following subsections, we present three case studies evaluating the effectiveness of our approach. For this purpose, we consulted human domain experts to specify search fields of interest. For every search field, we applied our proposed approach, first defining the search query and the appropriate time interval. A data set of web news articles was then collected from about 75 million different websites using webhose.io (Geva & Mor, 2019). The data was pre-processed, and strong signal candidates were generated. Based on the availability of the experts, the evaluation was carried out approximately 2 weeks after the results for the respective case study had been made available. A strong signal candidate is considered to be a strong signal if both the relevance and the novelty have been assessed as high (see in the section “Signal assessment”). The overall signal ratio is calculated as the proportion of all strong signal candidates that are not to be ignored, i.e., that have been considered to be of either high relevance or low novelty (or both).

### Case study 1: Bioeconomy

The term bioeconomy, sometimes also referred to as bio-based economy, describes the use of biotechnology to produce goods, services, or energy from biological material as a primary resource. This first search field thus describes one of the most-closely-observed emerging trends of the current decade. The keywords used to define it and the time period for data collection from October until December 2019 were specified with the assistance of human experts in this field. An overview of the analysis is given in Table 2.

The number of web news articles collected in the selected time frame (5938) already shows that there has been a high activity in this search field. After pre-processing, the remaining 5811 articles were passed on to the signal identification step, where they were annotated using NER and were then filtered down to 3194 weak signals. After the subsequent clustering and signal strength filter in the signal selection step, the experts were presented with 83 automatically-generated strong signal candidates for their assessment.

The evaluation by the experts shows that 30 (about 36%) of the generated candidates are considered strong signals. Together with those signals which have either a high relevance or high novelty, the signal ratio amounts to about 51%. Upon our investigation, the experts explained that the selected time frame (i.e., 3 months) reached too far into the past, and therefore much of the information had already been known before the analysis was conducted; this can also be seen from the overall frequency of signals having low novelty according to the cross table (45, corresponding to about 54%). This finding suggests that the time frame for the strong signal analysis should not be too long. Nevertheless, every second one of the candidates turned out to be a true signal.

Among the strong signals, our approach has signaled the topic “Texas Tech researchers testing new, innovative application for low-quality cotton” with a maximum number of 6 distinct sources on October 08, 2019. This identified signal relates to news reports about a company that was testing new and innovative applications for an existing biological material, and the experts evaluated it as highly relevant and highly novel. The distribution of the distinct sources of this signal consists of one source on October 07, 6 other sources on October 08, and only one other source on October 09. This specific development of the distinct sources over time shows a pattern that we have found for most of the strong signals in this search field. Almost all of the documents from different sources forming the signal are concentrated on a single day. It can therefore be assumed that it is quite likely to miss this and other highly-relevant and novel news if there is no early signal monitoring system in place which analyzes the documents published on every single day.

### Case study 2: AI startups

The second case study was meant to test the approach proposed in this paper for scouting for news related to startups in the field of artificial intelligence.

For this purpose, the search keywords were combined with the Boolean operator “AND” to increase the precision of the search field; however, only few such keywords were used. Moreover, based on the expert feedback received in the previous case study, the time frame for data collection was reduced to 1 month (namely, December 2019). Table 3 shows the overview of this analysis.

**Table 2:** Results of the case study on bioeconomy

Search field definition	Title	Bioeconomy				
	Search query	(“bio economy” OR “bioeconomy” OR “biopolymer” OR “biopolymers” OR “renewable resources” OR “renewable resource”)				
	Data sources	Web news articles				
	Time frame	3 months (October - December 2019)				
Data collection	Documents collected	5,938				
And pre-processing	Documents pre-processed	5,811 (97.86%)				
Signal identification	Weak signals	3,194 (54.96%)				
Signal selection	Number of clusters	857 (26.83%)				
	Strong signal candidates	83 (9.68%)				
Signal assessment	Frequencies			Relevance		
				Low	High	$\sum$
		Novelty	High	**8 (9.64%)**	**30 (36.14%)**	38 (45.78%)
			Low	41 (49.40%)	**4 (4.82%)**	45 (54.22%)
			$\sum$	49 (59.04%)	34 (40.96%)	83 (100.00%)
	Strong signals	30 (36.14%)				
	Signal ratio	50.60%				
Example strong signal	Topic	“Texas Tech researchers testing new, innovative application for low-quality cotton”				
	Signal strength	8				
	Maximum distinct sources	6 @ October 08, 2019				
	Distinct sources over time

**Table 3:** Results of the case study on AI startups

Search field definition	Title	AI startups				
	Search query	(“startup” AND “artificial intelligence”)				
	Data sources	Web news articles				
	Time frame	1 month (December 2019)				
Data collection	Documents collected	3,691				
and pre-processing	Documents pre-processed	3,687 (99.89%)				
Signal identification	Weak signals	2,195 (59.53%)				
Signal selection	Number of clusters	588 (26.79%)				
	Strong signal candidates	37 (6.29%)				
Signal assessment	Frequencies			Relevance		
				Low	High	$$\sum$$
		Novelty	High	**3 (8.11%)**	**23 (62.16%)**	26 (70.27%)
			Low	4 (10.81%)	**7 (18.92%)**	11 (29.73%)
			$$\sum$$	7 (18.92%)	30 (81.08%)	37 (100.00%)
	Strong signals	23 (62.16%)				
	Signal ratio	89.19%				
Example strong signal	Topic	“Intel buys Israeli AI chip startup Habana for $2B”				
	Signal strength	74				
	Maximum distinct sources	53 @ December 16, 2019				
	Distinct sources over time					

Within only one month 3691 news articles were found for the search query. After the automated processing of these documents, the experts were presented with 37 strong signal candidates after clustering.

The table of absolute and relative frequencies shows that 23 (approximately 62%) of the detected candidates have been evaluated as both highly relevant and highly novel, and they are therefore strong signals. It is recommended to pay immediate attention to them and to disseminate them to the relevant stakeholders (see in the section “Signal assessment”). The 7 signals with high relevance and low novelty (about 19%), and the 3 signals with low relevance but high novelty (about 8%) should also be given attention; they are to be watched and reviewed, respectively. The 4 signals with both low relevance and low novelty (about 11%) can be ignored. Overall, this results in a signal ratio of around 89%, which is considerably higher than in the previous case study.

The strong signal shown as an example in Table 3 (“Intel buys Israeli AI chip startup Habana for $2B”) concerns a widely-noticed merger and acquisition activity. Interestingly, the analysis of the signal’s distinct sources over time reveals that one news article each was published on December 03, 05, and 06, before this news reached its strongest peak on December 16 with 53 different sources. It then declined on December 17 to 14 different sources and finally to 2 additional sources each on December 18 and 19. However, the three articles published before the peak did not report on the acquisition itself, but rather on “advanced talks” about a possible acquisition. Despite the strength of the signal in the data sources it was previously unknown to the experts, and it would probably have remained undiscovered without our analysis.

### Case study 3: Low DC charging

In the third case study, signals in the area of low direct current (DC) charging for electric vehicles were to be identified. Since this search field is more specific than the previous ones, a larger number of keywords was needed and selected with the experts. We further set the time period to 2 months (namely, between January and February 2020), which lies between the durations used in the other two case studies. An overview of the results is given in Table 4.

The comparatively lower number of 1565 news articles found within 2 months reflects the high specificity of the search field. After data analysis, the experts were presented with 41 strong signal candidates. In this case, the experts’ assessment resulted in 19 strong signals (about 46%), which are considered highly relevant and novel. 15 candidates (approx. 37%) were of low relevance but high novelty, and no candidate was rated to be of high relevance but low novelty. In total, the signal ratio is approximately 83%, whereas 17% of the candidates can be ignored.

**Table 4:** Results of the case study on low DC charging

Search field definition	Title	Low DC Charging				
	Search query	(“cable monitoring” OR “cable guard” OR “50kW charger” OR “150kW charger” OR “cable failure” OR “cable safety” OR “cable damage” OR “broken cable” OR “burning cable” OR “cable maintenance” OR “Huber &Suhner” OR “Phoenix contact” OR “Mennekes” OR “ITT Inc” OR “battery maintenance” OR “DC charging” OR “smart charging” OR “smart cable” OR “smart battery” OR “fast charger systems”)				
	Data sources	Web news articles				
	Time frame	2 months (January - February 2020)				
Data collection	Documents collected	1,565				
and pre-processing	Documents pre-processed	1,565 (100.00%)				
Signal identification	Weak signals	1,030 (65.81%)				
Signal selection	Number of clusters	298 (28.93%)				
	Strong signal candidates	41 (13.76%)				
Signal assessment	Frequencies			Relevance		
				Low	High	$\sum$
		Novelty	High	**15 (36.59%)**	**19 (46.34%)**	34 (82.93%)
			Low	7 (17.07%)	**0 (0.00%)**	7 (17.07%)
			$\sum$	22 (53.66%)	19 (46.34%)	41 (100.00%)
	Strong signals	19 (46.34%)				
	Signal ratio	82.93%				
Example strong signal	Topic	“Total S.A. to install and operate 20,000 new EV charging points in Amsterdam”				
	Signal strength	13				
	Maximum distinct sources	6 @ Jan 22, 2020				
	Distinct sources over time					

The number of distinct sources of the exemplary topic “Total S.A. to install and operate 20,000 new EV charging points in Amsterdam” rose from 2 on January 21 to a peak of 6 on January 22, then declined to 4 on January 23 and to 1 on January 31. This signal was highlighted by the experts as particularly relevant, as the company mentioned therein, Total S.A., is a multinational integrated oil and gas company. This information is therefore seen as a strong signal that this company is increasingly investing in new business areas, in this case in the field of electromobility. It thus represents highly-important information for market and competitor monitoring. Interestingly, the experts classified the signal as novel, although the documents clustered in it had already been published in January, whereas the time frame of this search field reached until the end of February 2020.

## Comparison of clustering results for different linkage methods

Our approach makes use of the Ward linkage method, which minimizes the increase in the total within-cluster variance when merging two clusters, as described in the section “Signal selection”. Of course, choosing a different clustering approach or a different method for determining the threshold parameter for each corpus would have led to different results. While an infinite number of choices is possible for the latter, there is a manageable set of popular agglomerative clustering methods: single, complete, and average linkage.

The single linkage method measures the distance between clusters as the distance between their two closest objects. Since only one pair of objects thus needs to be close to merge two clusters, this approach is known to suffer from the chaining effect. Often, the result consists of one huge cluster, including highly different objects, as well as a number of very small clusters, encompassing only one or two objects.

In the complete linkage method, the distance between clusters is defined as the distance between their most distant objects. In contrast to single linkage this method typically results in more balanced clusters. Complete linkage is vulnerable to outliers, because objects very far from the cluster center can vastly influence the final output.

The average linkage method measures the distance between two clusters as the arithmetic mean of the distances between all pairs of elements, with one element chosen from each of the two clusters. Similar to Ward linkage, it is not sensitive to noise and leads to globular clusters (Manning et al., 2008).

In the following, we compare the results obtained for our three case studies using Ward linkage with the ones resulting from the other linkage methods. Our assessment focuses on the clustering and strong signal candidate detection part of the approach, because human assessment for a use case is not truly repeatable: an assessment conducted by an expert influences future assessments made by the respective person. Also, other parameters used in our case study, such as the threshold parameter determined per corpus, are kept constant across these evaluations.

For each one of our case studies, we compare the set of clusters resulting from Ward linkage, $\mathcal {C}_W = \{C_{W,1},..., C_{W, |\mathcal {C}_W|}\}$, with the one obtained for one of the other three methods average linkage (*A*), complete linkage (*C*) and single linkage (*S*), $\mathcal {C}_m = \{C_{m,1},..., C_{m, |\mathcal {C}_m|}\}$, where $m \in \{A, C, S\}$.

Evaluation metrics to be used for our analyses need to be agnostic to the cluster labels. Suitable metrics are Mutual Information, homogeneity, and completeness.

The Mutual Information is an entropy-based metric. Entropy measures the amount of uncertainty in a clustering $\mathcal {C} = \{C_1,..., C_{|\mathcal {C}|}\}$:

$$\begin{aligned} H(\mathcal {C}) = - \sum _{v=1}^{|\mathcal {C}|}P(C_v)\ln (P(C_v)), \end{aligned}$$

where $P(C_v)=\frac{|C_v|}{D}$ is the probability that a randomly-picked object from $\mathcal {C}$ falls into class $C_v$, and *D* is the total number of clustered elements. Building on this, Mutual Information (*MI*) between $\mathcal {C}_W$ and $\mathcal {C}_m$ is defined as

$$\begin{aligned} MI(\mathcal {C}_W, \mathcal {C}_m) = \sum _{v=1}^{|\mathcal {C}_W|} \sum _{k=1}^{|\mathcal {C}_m|} P(C_{W,v},C_{m,k}) \ln \left( \frac{P(C_{W,v},C_{m,k})}{P(C_{W,v})P(C_{m,k})} \right) , \end{aligned}$$

with $$P(C_{W,v},C_{m,k})=\frac{|C_{W,v} \,\cap \, C_{m,k}|}{D}$$.

Adjusted Mutual Information (*AMI*) additionally adjusts *MI* for chance:

$$\begin{aligned} AMI(\mathcal {C}_W, \mathcal {C}_m) = \frac{MI(\mathcal {C}_W, \mathcal {C}_m) - E[MI(\mathcal {C}_W, \mathcal {C}_m)]}{\max \{H(\mathcal {C}_W), H(\mathcal {C}_m)\} - E[MI(\mathcal {C}_W, \mathcal {C}_m)]}. \end{aligned}$$

The expected Mutual Information, featuring in the numerator and the denominator of this expression, can be dervied from a contingency table between the clustering results $\mathcal {C}_W$ and $\mathcal {C}_m$, with $n_{vk}=|C_{W,v} \cap C_{m,k}|$ denoting the number of elements falling into both $C_{W,v}$ and $C_{m,k}$. Moreover, $n_{v\cdot }=\sum _{k=1}^{|\mathcal {C}_m|} n_{vk}$ and $n_{\cdot k}=\sum _{v=1}^{|\mathcal {C}_W|} n_{vk}$ denote the row sums and the column sums, respectively. Based on this, we can obtain expected Mutual Information as

$$\begin{aligned}{} & {} E[MI(\mathcal {C}_W, \mathcal {C}_m)]=\sum _{v=1}^{|\mathcal {C}_W|} \sum _{k=1}^{|\mathcal {C}_m|} \sum _{n_{vk}=\max (1, n_{v\cdot }+n_{\cdot k}-D) }^{\min (n_{v\cdot }, n_{\cdot k})} \frac{n_{vk}}{D}\ln \left( \frac{ D \cdot n_{vk}}{n_{v\cdot } n_{\cdot k}}\right) \\{} & {} \quad \times \frac{n_{v\cdot }!n_{\cdot k}!(D-n_{v\cdot })!(D-n_{\cdot k})!}{D!n_{vk}!(n_{v\cdot }-n_{vk})!(n_{\cdot k}-n_{vk})! (D-n_{v\cdot }-n_{\cdot k}+n_{vk})!}. \end{aligned}$$

Due to the adjustment for chance, the values of *AMI* are more easily interpretable. While a value of 1 is attained if $\mathcal {C}_W$ and $\mathcal {C}_m$ have assigned the elements to clusters in an identical way, a value of 0 represents assignments of $\mathcal {C}_m$ that are not influenced by the assigments of $\mathcal {C}_W$. There can be negative values for assignments even less similar than those that can be expected to happen by chance (Vinh et al., 2009).

Homogeneity measures if the clusters solely contain members of one class:

$$\begin{aligned} G = 1 - \frac{H(\mathcal {C}_W|\mathcal {C}_m)}{H(\mathcal {C}_W)}, \end{aligned}$$

where the conditional entropy $H(\mathcal {C}_W|\mathcal {C}_m)$ is given by

$$\begin{aligned} H(\mathcal {C}_W|\mathcal {C}_m) = - \sum _{v=1}^{|\mathcal {C}_W|} \sum _{k=1}^{|\mathcal {C}_m|} \frac{n_{vk}}{D} \ln \left( \frac{n_{vk}}{n_{\cdot k}}\right) . \end{aligned}$$

Moreover, completeness measures if all class members of a respective cluster are assigned to the same cluster:

$$\begin{aligned} T = 1 - \frac{H(\mathcal {C}_m|\mathcal {C}_W)}{H(\mathcal {C}_m)}, \end{aligned}$$

where $H(\mathcal {C}_m|\mathcal {C}_W)$ is computed analogously to $H(\mathcal {C}_W|\mathcal {C}_m)$. The homogeneity and completeness measures can take values in the range [0, 1] (Rosenberg & Hirschberg, 2007).

The evaluation metrics were computed for three alternative linkage methods. The results are summarized in Table 5.

**Table 5:** Sensitivity analysis results

Linkage	*AMI*	Homogeneity	Completeness	Clusters
Case Study 1: Bioeconomy				
Ward	–	–	–	857
Average	0.585	0.700	0.983	214
Complete	0.742	0.860	0.982	442
Single	0.001	0.001	1	2
Case Study 2: AI Startups				
Ward	–	–	–	588
Average	0.614	0.705	0.984	198
Complete	0.741	0.836	0.985	335
Single	0.003	0.004	1	2
Case Study 3: Low DC Charging				
Ward	–	–	–	298
Average	0.665	0.758	0.994	298
Complete	0.779	0.867	0.989	198
Single	0.020	0.029	1	12

The results indicate that Ward linkage returns similar clustering results compared to the complete linkage method. Average linkage shows lower *AMI* scores than complete linkage, which suggests that the results for Ward linkage and complete linkage are more similar than the results for Ward linkage and average linkage.

The high completeness values can be explained by the smaller numbers of clusters for the average, complete, and single linkage methods. This is especially true in the case of single linkage, which has formed very few clusters.

The homogeneity values are high for average and complete linkage, although the clusters formed by these methods are bigger than the ones formed by Ward linkage. This means that completely homogenous clusters would not even be possible.

Single linkage shows very low similarity to the results of the Ward linkage method, as well as the expected behavior of chaining; in combination with agglomerative clustering and the used threshold parameter it has also led to the documents being assembled in very few, highly imbalanced clusters.

The next step in our approach is to identify weak signals coming from different sources (see in the section “Signal selection”), which are considered strong signal candidates; the remaining clusters and the documents associated with them are dropped. To compare the performance, we have carried out this step based on the clustering result of each of the different linkage methods. Our comparison is taking into account the documents included in the respective strong signal candidates. Specifically, the number of intersecting documents between the Ward linkage method results and the results for a different linkage method is used as an indicator for the similarity of the results.

Table 6 summarizes the results for the strong signal candidates regarding the different linkage methods.

**Table 6:** Comparison of strong signal candidates resulting for different linkage methods with the ones obtained for Ward linkage

Linkage	# Strong signal candidates	# Intersecting documents	Percentage
Strong signal candidates: Bioeconomy, total number of initial weak signals: 3194			
Ward	83	(889)	–
Average	7	348	39.1%
Complete	27	518	58.3%
Single	1	889	100%
Strong signal candidates: AI Startups, total number of initial weak signals: 2195			
Ward	37	(575)	–
Average	6	413	71.8%
Complete	4	340	59.1%
Single	1	575	100%
Strong signal candidates: DC Charging, total number of initial weak signals: 1030			
Ward	41	(352)	–
Average	2	105	29.8%
Complete	12	203	57.7%
Single	1	341	96.9%

The table shows that the complete linkage method still produces results most similar to the ones of Ward linkage: roughly $60\%$ of the documents associated with the strong signal candidates of Ward linkage are also part of the ones identified for complete linkage. In the AI Startups use case, these are 340 documents out of the 575 documents related to the strong signal candidates identified using the Ward method.

For average linkage, the results are less consistent over the different use cases. The AI Startups case shows a high percentage of intersecting documents (about $70\%$), while a rather low percentage (roughly $30\%$) is found for the DC Charging use case.

Overall, the strong signal results partially confirm robustness of the clustering approach used with regard to the linkage method chosen. The Ward and complete linkage methods produce fairly similar results. A change to the average linkage method however might produce different results, depending on the document corpus. Thus the clustering can be sensitive towards the change of the linkage method to average linkage.

The large number of clusters and strong signal candidates that the Ward linkage method creates is essential for our use case, because we want to be able to detect weak signals early on. Moreover, documents describing different new developments should be discriminated and allocated to different clusters to have the possibility of forming separate strong signal candidates. In our approach, we thus aim for clusters that only group very similar weak signals. The average linkage method might miss some of the relevant signals and might not be compatible with our overall approach in this respect, because it identifies fewer clusters (weak signals) and fewer strong signal candidates.

The single linkage method might perform better with a lower threshold parameter. However, the threshold parameter is determined individually for each data set in our approach. The similarities between documents can differ greatly between document corpora, and it would thus be improper to use the same threshold parameter for each of them. The threshold parameter itself ensures that documents in the cluster are not too distant from each other. Additionally, the chaining property of single linkage is unsuitable for our use case, because we want clearly demarcated, small and dense clusters with documents that revolve around a specific topic; the single linkage method would be more suitable to find single outliers, rather than strong signal candidates containing several weak signals pointing into the same direction.

 Clearly, the choice of *z* for the termination criterion for hierarchical clustering also has an impact on the clustering results. Recall from the section “Case Studies” that we set $z=0.05$. Keeping Ward as the linkage method fixed, setting $z=0.01$ results in 92.2%, 90.4% and 71.3% overlapping documents for case studies Bioeconomy, AI Startups and Low DC Charging, respectively, when compared to the clustering results obtained at $z=0.05$. With $z=0.1$, the documents overlap completely for all three case studies. Therefore, we consider the results for different values of *z* to be sufficiently similar.

## Discussion

In the following subsections we will first discuss the findings derived from our three case studies, and we will then describe some limitations of our approach, together with future research opportunities.

### Findings

In the three case studies presented, the 11,194 documents collected were condensed and filtered to a mere 161 strong signal candidates. While human experts could hardly scan all the documents, the high level of reduction achieved makes it feasible for them to check the automatically-generated signals, boosting their efficiency. Furthermore, the relatively small proportion of strong signal candidates among the many clusters (1743 in total) indicates that our assumption of a highly right-skewed distribution of the signal strengths, as discussed in the context of Equation (6), is correct.

Although there are existing approaches tracking topics of interest over time as well, e.g., trending hashtags in Twitter (Aiello et al., 2013; Bello-Orgaz et al., 2014; Xie et al., 2016) or trends in patent databases (Milanez et al., 2014; Momeni & Rost, 2016; Noh et al., 2016), these approaches make use of only one or just a few sources. In contrast to this, our approach uses a large number of different sources (about 75 million websites, see in the section “Case studies”). This allows us not only to identify signals at scale, but also to automatically prioritize them (see in the section “Signal selection”) before presenting them to human experts. By separating noise from actual news, companies can change the way in which they deal with the increasing information overload in their corporate environment, and they are thus able to save a lot of valuable time and human resources.

The analysis of the distinct sources over time in the case studies has also shown that the attention span for the identified topics is often concentrated around one day. Consequently, it is reasonable to assume that the probability of missing relevant news is quite high; this risk can be reduced with our approach.

The domain experts assessed 109 (about 68%) of the 161 strong signal candidates as either highly relevant or highly novel (or both); 72 (about 45%) of them were assessed as both highly relevant and novel, and were thus considered to be strong signals. These high true positive rates suggest that our proposed approach can be used as a precise early warning system. They also indicate that the fact checked by the approach (namely, that there are several weak signals coming from different sources in a short period of time) may indeed be deemed a good indicator for the emergence of a strong signal.

Overall, 63 (around 39%) of the strong signal candidates turned out not to be novel to our experts. At around 54% the fraction already known was especially high in our first case study, where we had analyzed data from the past three months. Such a long time frame may thus bear the risk of detecting developments that have already come to the experts’ attention. Shorter back periods seem advisable, and this is why we went with one month and two months, respectively, for the following case studies. To some extent, the observed percentage of strong signal candidates with a low novelty may also have been driven by the approximately two-week time lag between the provisioning of the results and the evaluation by the experts. The sooner the strong signal candidates are disseminated and processed further, the higher the degree of novelty that can be expected.

The feedback from the experts concerning the case studies has shown that our proposed approach is useful for the following (non-exhaustive) list of use cases: competition and market monitoring (competitive intelligence); identification of companies, potential partners and customers (company scouting); identification of startups (startup scouting); identification of key areas of research; detection of new topics (trend scouting) and new technologies (technology scouting); gaining knowledge about new product developments and business cooperations; support in calculation of business cases; development of the corporate strategy; and the further development of the existing portfolio. The preferred interval at which new strong signal candidates should be presented to the experts ranges from weekly over bi-weekly to monthly. With regard to the promising results of the third case study, it would be possible, for example, to implement a bi-weekly notification interval within a two-month analysis period using a moving window approach. Possible duplicates of previously-reported strong signal candidates could be eliminated based on similarity comparisons of the respective cluster centers.

The theory behind detecting early signals of change has often been criticized in the literature for various reasons: Due to their low visibility, as well as their lack of precision and confirmation, it has been assumed that they are often overlooked or even ignored due to existing human mental models and the related experience bias (Cawley & Talbot, 2010; Mühlroth & Grottke, 2018). It has further been argued that it is not enough to detect a signal once; rather, a collection of them is required to eventually point towards a more concrete signal (Kuosa, 2010; Hiltunen, 2010; Saritas & Smith, 2011). In addition, Thorleuchter et al. (2014) have concluded that supervised machine learning fails for the detection of weak signals because of their low frequency and the resulting small number of positive training examples. On the one hand, the outcomes of our case studies indicate that it may indeed be due to the experience bias that early signals of change are more difficult to detect without data-driven support; otherwise, the domain experts would have rated the novelty of our results lower. On the other hand, the fact that we were able to detect such novel signals also shows that the careful combination and application of unsupervised machine learning techniques is able to reduce the above-mentioned risks.

We therefore recommend companies to establish this or a similar approach as a continuous tool for monitoring strategically-important search fields.

### Limitations and future research opportunities

Despite the encouraging results of the case studies and the interesting implications derived from them, there are also limitations of our approach. We describe them below, and we also propose opportunities for future research.

First, it should be noted that, despite the increased degree of automation in our approach, human expert involvement is still required. Since there are no generally-accepted data sets for the detection of early signals of change, companies have to collect the data by themselves, and search queries need to be defined manually. However, once the query has been defined, the approach can be applied in a fully-automated fashion. Future research in this area could focus on query creation, data acquisition and signal assessment by supporting artificially-intelligent systems.

Second, the selection, combination, and parameterization of the unsupervised machine learning techniques used may be subject of discussion. Although we have carefully applied them in line with the current state of research, to some extent our approach still incorporates our own beliefs about the data and the results. Specifically, the substantial restriction of the allowed classes for NER (see in the section “Signal identification”) and the threshold applied during clustering (see in the section “Signal selection”) may be regarded as subjective to a certain degree. However, the results obtained in our case studies have indeed proven to be of added value to the human experts. Further research in this area could focus on making the proposed approach even more robust.

Third, there are several indicators that point to the strength of a signal. In our proposed approach, we have focused on one possible, purely quantitative indicator, namely the count of a signal’s (i.e., a cluster’s) unique document sources (i.e., distinct website domains). This indicator implements the idea that, if a signal is found in different sources over a short period of time, a formerly weak signal has now received increasing attention and thus strengthened (see also in the section “Theoretical framework”). As a total of 109 (about 68%) of the strong signal candidates indicated by our approach were rated as either highly relevant, highly novel, or both by the domain experts, we consider our proposed approach as an effective early warning system.

Fourth, we cannot claim that our approach has detected all existing strong signals, and we have not been able to evaluate the false-negative rate. Indeed, this is hardly possible since no information on the true population of strong signals is available. As not all strong signals develop into a trend, even a retrospective analysis based on an exhaustive list of all trends that emerged subsequently would fail to identify all strong signals in the observation period; it would therefore allow the calculation of neither the false-negative rate nor the true-positive rate. With the research method applied we have at least been able to evaluate the latter one, and we have avoided the risk of a look-ahead bias immanent to a retrospective analysis.

Finally, although our approach can detect strong signals of change, their dissemination and their further tracking is not part of our proposed approach. Future research could therefore address the question of how the strong signals detected can be continuously monitored and analyzed for potentially-emerging trends.

## Conclusion

This paper was motivated by the lack of data-driven tools and automation to support the early identification of relevant signals of change for companies. To this end, we have developed an innovative approach that leverages unsupervised machine learning techniques to enhance corporate strategy, foresight, and innovation processes.

Our proposed innovation signals triple diamond model is based on the recent state of the art in this field and has been designed to achieve the highest-possible degree of automation for its purpose. After one or more search fields of interest have been defined and the data has been collected and pre-processed, Named Entity Recognition is applied to focus on information related to a natural or legal person. The weak signals obtained are then clustered and analyzed for their signal strength, are prioritized and filtered by an automated selection procedure, and are then presented to the human expert as candidates for strong signals. Finally, after having been assessed for their relevance and novelty, they are disseminated to subsequent business processes for further consideration.

To demonstrate the effectiveness of our approach, we have evaluated it by means of three independent case studies. In addition, we evaluated the robustness of our approach by performing a sensitivity analysis. Our results indicate that in all cases a large proportion of the signal candidates identified were indeed highly-relevant and highly-novel strong signals of change, that we were able to counteract the human experience bias, and that our approach has the potential to save valuable time and human resources due to its increased degree of automation. Therefore, we recommend companies to establish such an approach for continuously monitoring their corporate environment for early signals of change more effectively and efficiently.

With this paper, we hope to have made a significant contribution to research and practice in order to support better-informed decision making for the benefit of technology and society.

## Data Availability

The authors declare that data and material can be made available on request.

## Code availability

The authors state that the code can be made available on request.
